# Diagnosis of cutaneous tuberculosis (lymph node scrofuloderma) using the Xpert MTB/RIF® method^☆^^☆☆^

**DOI:** 10.1016/j.abd.2020.01.009

**Published:** 2020-11-19

**Authors:** Lilian Lemos Costa, John Verrinder Veasey

**Affiliations:** Dermatology Clinic, Santa Casa de Misericórdia de São Paulo, São Paulo, SP, Brazil

**Keywords:** Diagnosis, Diagnostic tools, Mycobacterium tuberculosis, Polymerase chain reaction, Rifampicin, Tuberculosis, cutaneous

## Abstract

Cutaneous tuberculosis is a rare infection that is difficult to diagnose, because it shows less sensitivity and specificity in classic complementary exams when compared with the pulmonary form. The Xpert MTB/RIF® method offers an early diagnosis that identifies the DNA of *Mycobacterium tuberculosis* and the main mutations that give the bacterium resistance to rifampicin. The authors present a case of scrofuloderma whose diagnosis was quickly obtained through the secretion of a cervical lesion, allowing an early diagnosis and the initiation of appropriate treatment.

Cutaneous tuberculosis (CTB) accounts for approximately 1%−2% of tuberculosis cases.^1^ Scrofuloderma is the most common form of CTB in developing countries such as Brazil, characterized by subcutaneous nodules with fistulas and secretion output.^1^ The diagnosis of tuberculosis is confirmed by evidence of the bacillus, usually *Mycobacterium tuberculosis* (MTB), a challenging situation for cases of CTB, since the classic diagnostic methods have less sensitivity and specificity for skin presentations in relation to the pulmonary form.^2^ This led the World Health Organization (WHO) to recommend the use of polymerase chain reaction (PCR) in the diagnosis of this infection; the Xpert MTB/RIF® is one of the devices indicated.^3^, ^4^ It is an automated, fast, semi-quantitative PCR method that simultaneously detects microorganisms of the MTB complex and the resistance of the agent to rifampicin in liquid clinical samples over a period of two hours. While the use of this method in samples of pulmonary tuberculosis materials is well established, its use in extrapulmonary infections has been poorly described.^3^, ^4^, ^5^, ^6^, ^7^

The authors report the case of a 19-year-old man, born in Luanda, Angola and living in São Paulo, Brazil for one year, who presented painful nodules with progressive growth for five months. He denied any systemic or respiratory symptoms. On physical examination, nodules and abscesses were noted on the cervical and thoracic regions, with fistulization and discharge of secretion (Fig. 1). He brought previous exams to the consultation, indicating non-reactive serology for hepatitis B, C, HIV, and HTLV 1 and 2, as well as a chest X-ray with no evidence of pulmonary involvement. The authors opted for puncture and aspiration of one of the cervical lesions for diagnostic analysis with Xpert MTB/RIF®, which showed a positive result for the presence of a rifampicin-sensitive MTB strain (Figure 2, Figure 3).

**Figure 1:**
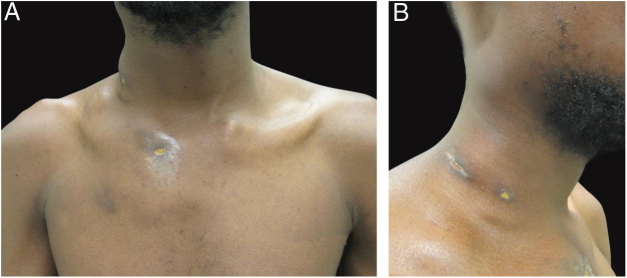
Clinical aspect of the patient. (A − B) dermatosis located on the right side of the cervical and upper thoracic regions characterized by cold and painless abscesses up to 5 cm in diameter, some with fistulization and ulcers.

**Figure 2:**
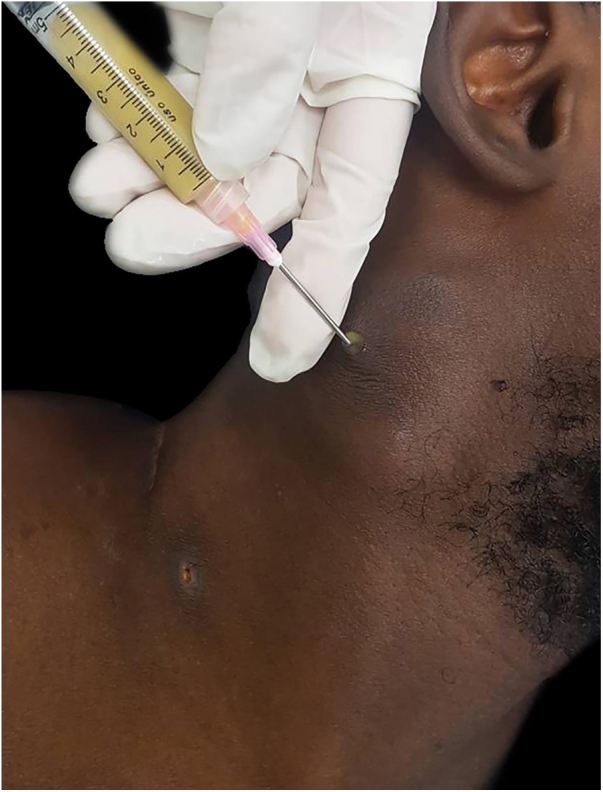
Collection of cervical abscess material by puncture with a thick needle.

**Figure 3:**
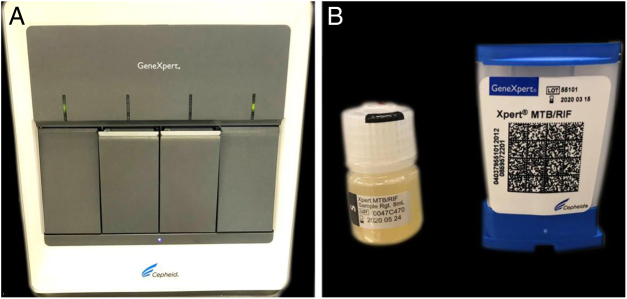
Materials for analysis by the Xpert MTB/RIF® method. (A), GeneXpert® device. (B), Buffer solution for treatment of the sample and cartridge for containing the material and reagents to be processed in the device.

While samples of pulmonary tuberculosis (sputum) show a sensitivity of 89% with the referred method, the sensitivity of the analysis of lymph node aspirate reaches 97%.^3^, ^5^, ^8^ In a study conducted in Ethiopia, 15 cases of lymph node aspirate were analyzed by Xpert MTB/RIF®, being positive in 33% of cases, while fluorescence microscopy showed the bacillus in only 6.7%.^6^ Another meta-analysis study indicated a different sensitivity for samples from different locations, with 83.1% in puncture of scrofuloderma, 80.5% in meningoencephalic tuberculosis, and only 46.4% in pleural fluid.^9^

Another relevant finding obtained by this method is information about the resistance of the bacillus to rifampicin, a bactericidal drug of great importance in the treatment of CTB. It is estimated that currently about 3.5% of new tuberculosis cases and 18% of previously treated cases are caused by rifampin-resistant MTB.^10^

Ideally, a PCR exam for the detection and identification of MTB should be accessible to most healthcare services in Brazil. Some already have the Xpert MTB/RIF® method for analyzing sputum samples, which can also be used to analyze other liquid materials. The present cases illustrates the benefit of this method in obtaining a rapid diagnosis using material from puncture and aspiration of a lymph node; the identification of antimicrobial sensitivity to rifampicin allowed an early treatment.

## Financial support

None declared.

## Authors’ contributions

Lilian Lemos Costa: Approval of the final version of the manuscript; drafting and editing of the manuscript; collection, analysis, and interpretation of data; intellectual participation in propaedeutic and/or therapeutic conduct of studied cases; critical review of the literature; critical review of the manuscript.

John Verrinder Veasey: Approval of the final version of the manuscript; design and planning of the study; drafting and editing of the manuscript; collection, analysis, and interpretation of data; effective participation in research orientation; intellectual participation in propaedeutic and/or therapeutic conduct of studied cases; critical review of the literature; critical review of the manuscript.

## Conflicts of interest

None declared.
